# Case Report on Spontaneous Uterine Rupture in the Early Second Trimester: A Rare but Life‐Threatening Event

**DOI:** 10.1155/crog/9949364

**Published:** 2026-04-23

**Authors:** Minh Tam Le, Ngoc Anh Thi Vo, Duy Trong Tran, Thanh Huyen Thi Ho, Vu Quoc Huy Nguyen

**Affiliations:** ^1^ Department of OBGYN, Hue University of Medicine and Pharmacy, Hue University, 06 Ngo Quyen Street, Thuan Hoa District, Hue, 49120, Vietnam, hueuni.edu.vn

**Keywords:** placenta accreta, pregnancy, uterine rupture

## Abstract

Spontaneous uterine rupture during the first half of pregnancy is extremely uncommon and presents significant diagnostic and therapeutic challenges. This case report details a 27‐year‐old gravida 3 para 1 woman at 14 weeks of gestation who experienced acute abdominal pain and hypovolemic shock. Despite the absence of previous uterine surgery, imaging revealed a significant hemoperitoneum, and subsequent emergency laparoscopic and laparotomy validated a uterine fundal rupture attributable to placenta accreta spectrum (PAS). The uterus was successfully preserved after surgical repair. Histopathological examination confirmed localized myometrial infiltration by placental tissue, consistent with PAS. This report emphasizes the necessity of including PAS–related uterine rupture in the differential diagnosis of acute abdomen during early pregnancy, even when classical risk markers are absent. The example underscores the need of timely surgical intervention in securing positive mother outcomes and maintaining reproductive potential. The postoperative course was uneventful, and the patient was discharged in stable condition with preservation of uterine integrity. This article seeks to enhance clinical knowledge and promote vigilance among obstetricians and emergency physicians on early‐pregnancy uterine rupture by contributing to the scarce literature on the subject.

## 1. Introduction

Uterine rupture constitutes a critical emergency in obstetrics, marked by the structural breakdown of the uterine wall, which can swiftly result in significant hemorrhage and maternal instability. While the majority of occurrences occur in the context of a previously scarred uterus, instances of rupture in an unscarred uterus have also been documented, though significantly less often [1]. Population‐based studies from affluent regions indicate that uterine rupture is a rare occurrence (one in 1536 births), with reported rates fluctuating significantly based on obstetric practices and maternal risk factors. The primary rupture of an unscarred uterus is exceptionally uncommon, occurring in around one in 10,000 to one in 15,000 births [1].

Prior surgical interruption of the uterine wall, particularly cesarean section, is universally recognized as the most significant predisposing factor for uterine rupture [1, 2]. Additional problems linked to compromised myometrial integrity, obstructed labor, grand multiparity, aberrant fetal presentation, instrumental delivery, and gestational trophoblastic illness or abnormal placentation (placenta accreta spectrum [PAS] disorders) may further elevate this risk. Other recognized risk factors for uterine rupture are labor induction or augmentation with prostaglandins or oxytocin, fetal macrosomia, and obstructed labor.

Unlike rupture during labor or late gestation, uterine rupture in early pregnancy is exceptionally rare and so often undetected. The ambiguous clinical presentation frequently leads to diagnostic delays, especially in women lacking traditional obstetric risk factors. We report an uncommon instance of spontaneous uterine rupture at 14 weeks of gestation due to placenta accreta in a 27‐year‐old woman (G3P1) with no prior cesarean section history.

## 2. Case Presentation

The clinical course is summarized chronologically from initial symptom onset to postoperative recovery. A 27‐year‐old woman, gravida 3 para 1‐0‐1‐1, presented to the emergency department at 14 weeks and 2 days of gestation with a sudden onset of severe lower abdomen pain. Her obstetric history included one previous vaginal delivery and a tubal ectopic pregnancy managed with laparoscopic salpingectomy 3 years earlier. She had no history of uterine surgery, dilatation, curettage, or any other intrauterine procedures. Her personal and family medical history was unremarkable.

The patient attended her first prenatal visit at 8 weeks of gestation. The first‐trimester ultrasound and aneuploidy screening were unremarkable. At 13 weeks, she began experiencing mild hypogastric pain that persisted for 1 week, which acutely worsened 1 day prior to admission.

Upon arrival, she exhibited hypovolemic shock characterized by pallor, hypotension (blood pressure 70/40 mmHg), and a heart rate of 70 beats/min. The physical examination revealed diffuse abdominal tenderness with guarding and rebound soreness. Laboratory investigations showed a rapid decline in hemoglobin from 94 to 77 g/L. Transabdominalultrasound demonstrated a viable intrauterine pregnancy consistent with 14 weeks of gestation, a closed cervix, a hematoma adjacent to the left uterine wall, and a moderate volume of free intraperitoneal fluid. Computed tomography (CT) revealed difficulty visualizing the ovaries, large clots surrounding the uterus extending into the left paracolic gutter, and moderate‐to‐large volume hemoperitoneum. Due to rapid clinical deterioration, further imaging, such as MRI, was not feasible prior to surgical intervention. The absence of detailed preoperative imaging findings in this case (MRI) reflects the emergency clinical context rather than a diagnostic limitation, as definitive confirmation of PAS was established by histopathological examination.

**Figure 1:**
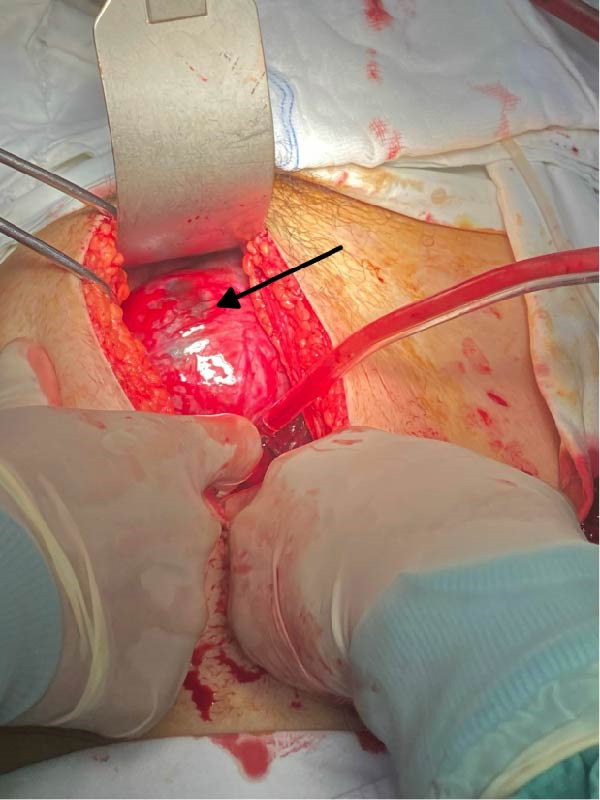
Intraoperative view during emergency laparotomy showing the posterior uterine fundus. The myometrial wall appears markedly thinned with loss of normal muscular architecture and prominent subserosal vascularity is visible over the fundal surface. These findings raised strong intraoperative suspicion of abnormal placental adherence at the site of uterine rupture.

**Figure 2:**
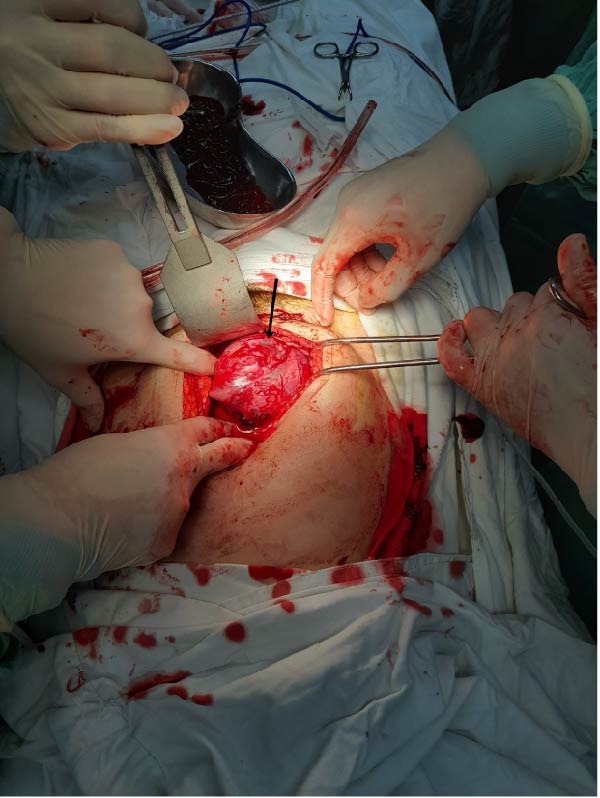
Intraoperative photograph demonstrating a focal defect at the posterior uterine fundus (arrow), corresponding to the site of uterine rupture. The surrounding myometrium is extremely thin with exposed subserosal vessels, consistent with structural weakening of the uterine wall at the placental implantation site.

**Figure 3:**
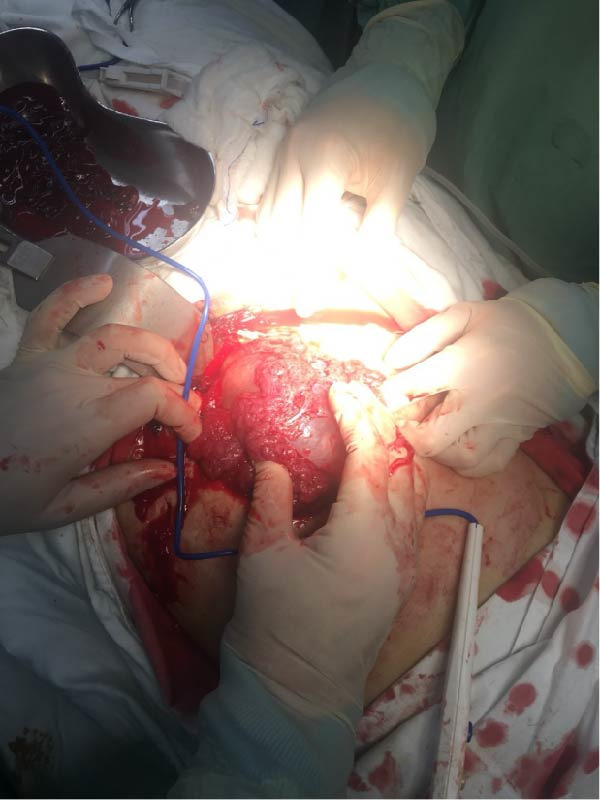
Gross specimen of the placenta following surgical removal. The placental tissue appears irregular and friable, with focal areas suggestive of deep attachment to the underlying myometrium. These macroscopic features were considered abnormal and prompted further histopathological evaluation.

Diagnostic assessment focused on identifying the source of hemoperitoneum in the setting of early pregnancy and hemodynamic instability. The patient received immediate resuscitative measures, including intravenous fluids and blood transfusion. Although the patient presented with hemodynamic instability, an initial laparoscopy was performed as a rapid diagnostic approach to identify the source of intra‐abdominal bleeding. Diagnostic laparoscopy revealed a massive hemoperitoneum with approximately 1500 mL of fresh blood and a large pelvic hematoma. Due to massive hemoperitoneum and ongoing bleeding, immediate conversion to laparotomy was undertaken to ensure effective hemostasis. Conversion to laparotomy was performed, yielding an additional 500 g of clotted blood. Surgical management aimed to achieve rapid hemostasis while preserving uterine anatomy whenever feasible. Intraoperative view of the posterior uterine fundus demonstrating a diffusely thinned myometrial wall, and active bleeding was noted from the posterior aspect, suggestive of uterine rupture associated with abnormal placental invasion (Figure 1). A markedly thinned posterior uterine fundus with visible subserosal vasculature and a focal active bleeding point, consistent with uterine rupture associated with placenta accreta (Figure 2). The fetus and placenta were removed, and the uterine defect was repaired in layers to achieve hemostasis. Gross appearance of the placenta after surgical removal, showing irregular placental tissue with focal areas of deep myometrial invasion, consistent with PAS disorder. The abdominal cavity was irrigated and inspected for additional bleeding (Figure 3).

**Figure 4:**
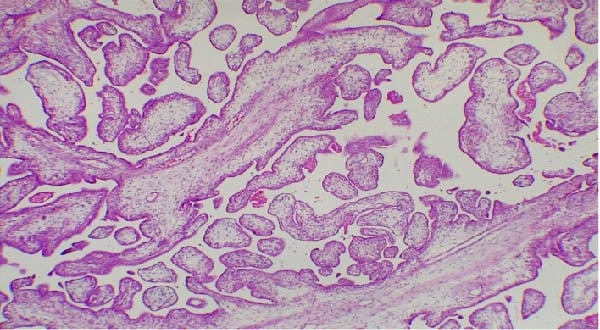
Histopathological examination of the placental tissue (hematoxylin and eosin stain, ×100) demonstrates chorionic villi in direct contact with and focally invading the myometrium, in the absence of an intervening decidual layer. These findings are consistent with placenta accreta spectrum.

Intraoperative transfusion included three packed red blood cell units and two fresh‐frozen plasma units. Postoperative recovery was uneventful, and the patient was discharged on postoperative day 5. Histopathological examination demonstrated chorionic villi in close apposition to the myometrium with focal absence of the decidual layer, without clear evidence of deep myometrial invasion. These findings are suggestive of abnormal placentation consistent with a suspected early form within the PAS (H&E stain, magnification ×100; Figure 4). The postoperative course was uneventful, and the patient was discharged in stable condition with preservation of uterine integrity.

## 3. Discussion

This case report was prepared in accordance with the CARE (case report) guidelines. Uterine rupture during the first half of pregnancy is extremely rare and often difficult to diagnose due to its nonspecific presentation. FIGO consensus guidelines on PAS emphasize that PAS is a growing obstetric concern with significant maternal morbidity, requiring accurate prenatal diagnosis and multidisciplinary management. The guidelines highlight the limitations of current imaging approaches, the need for standardized diagnostic criteria, and the importance of expertise in improving detection and outcomes [3]. PAS disorders are characterized by abnormal anchoring of placental tissue to the uterine wall, resulting from defective decidualization at the implantation site. This pathological adherence can weaken the underlying myometrium and predispose the uterus to rupture, even in early gestation [4]. Although typically associated with previous uterine instrumentation, PAS–related uterine rupture in the first half of pregnancy has been reported even in women with an unscarred uterus.

Imaging assessment of PAS during the first trimester remains challenging. Sonographic markers that are well described in later gestation may be absent or subtle early in pregnancy, reducing diagnostic confidence [5]. Advanced imaging, such as MRI, may provide additional information in selected cases, although its use is often constrained in emergency settings [6, 7]. As a result, clinicians may not consider PAS‐related rupture in early gestation, potentially delaying diagnosis—as seen in the current case.

Although prior cesarean delivery remains the dominant risk factor for uterine rupture, alternative mechanisms should be considered in women without uterine scars. In such cases, localized myometrial weakness and abnormal placental invasion may play a critical role [8, 9]. Our patient did not have a prior cesarean or intrauterine instrumentation, but she had undergone laparoscopic salpingectomy for ectopic pregnancy. Several reports suggest that salpingectomy—particularly when performed using monopolar or bipolar electrosurgery—may compromise adjacent myometrial integrity and contribute to uterine rupture in subsequent pregnancies [10, 11]. The mechanism may involve thermal injury leading to fibrosis, thinning, and reduced elasticity of the uterine wall [12, 13]. Moreover, the localized myometrial damage may impair vascular development and decidualization, predisposing to abnormal placental implantation [14]. These mechanisms likely explain the occurrence of PAS and uterine rupture in our case.

Subsequent pregnancies after prior mid‐trimester uterine rupture appear to carry an increased risk of PAS and recurrent uterine rupture, although no maternal deaths have been reported. While fetal outcomes are generally favorable, further studies are needed to better characterize maternal and fetal risks in this rare clinical scenario [15]. In women presenting with abdominal pain and signs of acute intra‐abdominal bleeding, immediate hemodynamic resuscitation and prompt identification of the source are essential. In stable pregnant patients, the peritoneal fluid alone is not always pathological [16]. However, fluid with signs of hemodynamic compromise should raise concern for rupture or other acute abdominal pathology. In early pregnancy, differential diagnoses may include ruptured ovarian cysts or heterotopic pregnancy [17]. Our patient had no ovarian pathology on prior imaging and had a confirmed intrauterine gestation, making such causes less likely.

Delayed diagnosis in this case may have been due to the gradual erosion of the myometrium by invasive trophoblasts, leading to slow accumulation of hemoperitoneum, transient peritoneal irritation, and minimal early hemodynamic changes. Spontaneous peritoneal fluid reabsorption over several days may mask signs of active bleeding [18].

In such diagnostically challenging cases, laparoscopy serves both diagnostic and therapeutic purposes. Minimally invasive surgery is considered safe during pregnancy when appropriately indicated and performed by experienced teams [19, 20]. In our case, due to significant hemoperitoneum and rapid clinical deterioration, laparoscopy followed by conversion to laparotomy was justified.

PAS–related uterine rupture is hazardous due to the increased neovascularization at the implantation site, resulting in more extensive hemorrhage compared to rupture at cesarean scar sites [9]. While hysterectomy is often the definitive treatment for uncontrollable bleeding, conservative approaches—including placental removal, uterine repair, adjuvant methotrexate therapy, and uterine artery ligation—have been reported in selected cases [21, 22]. However, some data suggest that conservative management may carry up to a fourfold increase in maternal mortality risk compared to hysterectomy [4]. Notably, early laparoscopic or surgical exploration is recommended when definitive imaging findings are absent. Timely surgical intervention is crucial to minimize maternal morbidity and mortality [23]. In our case, with effective hemostasis, uterine conservation was successful, and the patient was discharged without complications.

## 4. Conclusion

Spontaneous uterine rupture during the early second trimester is an exceedingly rare but potentially life‐threatening complication that may occur in the setting of PAS disorders, even in the absence of a uterine scar. Early diagnosis remains challenging due to nonspecific symptoms and the limited sensitivity of imaging modalities in early pregnancy. A high index of clinical suspicion is required, particularly in patients with risk factors such as prior pelvic surgery.

The findings suggest abnormal placental adherence rather than definitive invasive PAS, highlighting the diagnostic challenges in early pregnancy. Prompt surgical exploration—especially when imaging is inconclusive—can be life‐saving. Conservative surgical management may be feasible in hemodynamically stable patients if bleeding can be controlled, allowing for uterine preservation and favorable postoperative outcomes.

## Author Contributions

**Minh Tam Le:** conceptualization, methodology, investigation, project administration, supervision, funding acquisition, writing – original draft, writing – review and editing. **Ngoc Anh Thi Vo:** data curation, investigation, writing – original draft. **Duy Trong Tran:** data curation, methodology, investigation, writing – review ad editing. **Thanh Huyen Thi Ho:** data curation, investigation, writing – review and editing. **Vu Quoc Huy Nguyen:** conceptualization, supervision, investigation, writing – review and editing.

## Funding

The authors also acknowledge partial support from the Hue University through the Core Research Program (Research Group on Reproductive Medicine, Grant NCTB.DHH.2025.07).

## Disclosure

The sponsor had no role in the design, execution, analysis, or interpretation of the study results.

## Ethics Statement

Authors confirm that this work was approved by the Hue University of Medicine and Pharmacy Ethics Committee (Approved Number H2026/050). Patients all agree to participate with a consent form.

## Consent

Written informed consent was obtained from the patient for publication of this case report and accompanying images.

## Conflicts of Interest

The authors declare no conflicts of interest.

## Data Availability

The dataset used and/or analyzed during the current study is available from the corresponding author upon reasonable request.
